# Finite Element Prediction of Residual Stress and Deformation Induced by Double-Pass TIG Welding of Al 2219 Plate

**DOI:** 10.3390/ma12142251

**Published:** 2019-07-12

**Authors:** Abdulrahaman Shuaibu Ahmad, Yunxin Wu, Hai Gong, Lin Nie

**Affiliations:** 1 College of Mechanical and Electrical Engineering, Central South University, Changsha 410083, China; 2 State Key Laboratory of High-Performance Complex Manufacturing, Central South University, Changsha 410083, China; 3 Light Alloy Research Institute, Central South University, Changsha 410083, China

**Keywords:** TIG welding, residual stress, deformation, Al 2219, FE model, DFLUX subroutine

## Abstract

Finite element (FE) analysis of welding residual stress and deformation is one of the essential stages in the manufacturing process of mechanical structures and parts. It aids in reducing the production cost, minimizing errors, and optimizing the manufactured component. This paper presents a numerical prediction of residual stress and deformation induced by two-pass TIG welding of Al 2219 plates. The FE model was developed using ABAQUS and FORTRAN packages, Goldak’s heat source model was implemented by coding the nonuniform distributed flux (DFLUX) in user subroutine to represent the ellipsoidal moving weld torch, having front and rear power density distribution. Radiation and convection heat losses were taken into account. The mechanical boundary condition was applied to prevent the model from rotation and displacement in all directions while allowing material deformation. The FE model was experimentally validated and the compared results show good agreement with average variations of 18.8% and 17.4% in residual stresses and deformation, respectively.

## 1. Introduction

Tungsten Inert Gas (TIG) welding is a commonly used manufacturing process by the automobile, aviation, and aerospace manufacturing industries, etc. for joining non-ferrous metals [1] such as aluminum and magnesium. The induced thermal cycle from the TIG welding results in high residual stresses and deformation on the weld metal and heat-affected zone (HAZ), which adversely affects the mechanical performance of the components. Therefore, they require a significant consideration during design and manufacture of structural elements. Welding residual stress and distortion develop as a result of local plastic deformation introduced due to rapid heating followed by the subsequent uncontrolled cooling phase [2]. The weld metal is subjected to differential volumetric expansion and contraction at both macroscopic and microscopic level. The macroscopic volumetric changes take place during cooling due to metallurgical transformation. Residual stress as high as the yield stress of the base material could develop at the fusion zone (FZ) and HAZ [3,4,5]. There are various factors and parameters influencing the magnitude and distribution of residual stresses and deformation during welding. They include the type of weld, number of weld-passes, sequence and direction of welding stitches, fixing method, etc.

Furthermore, weld metals experience changes in mechanical and physical properties due to the effect of the weld thermal cycle. As the temperature of the weld area rises, the yield strength and elastic modulus of the material decreases, while specific heat and thermal expansion increases. This affects shrinkage and deformation of the material, as well as the heat flow and uniformity of heat distribution [6]. Residual stress at weld joint influences fatigue failure, brittle fracture, hot cracking of the weld zone, initiation and propagation of cold cracks and fatigue cracks at ambient temperature. In addition, corrosion cracking occurs, which lead to rapid failure of weld joint in a corrosion environment [7]. The aforementioned effects attract a significant cost of repairs and restoration of components [8].

Thus, analysis of residual stress is regarded as one of the compulsory stages required in the design and manufacture of components, which help in estimating and analyzing their reliability under various loading conditions. Numerical simulation using finite element methods (FEM) is the commonly used technique to predict and analyze welding residual stresses [9], which is less time-consuming, cost-effective and offers greater versatility compared to experimental measurements [1]. FE simulation of welding process using Goldak’s ellipsoidal heat source model has been applied to predict residual stresses and deformation, which results from the induced transient thermal field by many researchers, and the experimental validation shows good agreement [5,10,11].

Rong et al. consider the effect of non-linear yield stress curves, and multi-constraint equation to study residual stress and distortion, resulting from metal active gas welding of S355JR steel using FEM. The experimentally validated results show good agreement with the FEM [5]. Obeid et al. analyzed the thermal and residual stress field induced by TIG welding of line pipe. FE simulation of single pass weld overlay and girth welding was performed using ABAQUS user subroutine code. A moving heat source based on Goldak’s ellipsoid heat source model was used. The FEM was validated using previous experiments of the same sections of carbon-manganese C-Mn steel pipe lined with stainless steel, and the results show good agreement [10]. Almeida et al. performed a TIG butt-welding simulation on thin AISI 316L stainless steel plates, the induced residual stresses due to the double ellipsoidal heat source are found to be in good agreement with the results obtained from experiment [2]. Venkata et al. performed an FE simulation of three-pass TIG welding to predict the transient-thermal profile and residual stress [11]. Element activation and deactivation were used to represent the deposition of weld bead in the heat transfer analysis, and the results were verified using incremental deep-hole drilling and neutron diffraction method, which were reported to be in good agreement.

Moreover, Artificial Neural Network (ANN) has been employed to simulate the welding process and predict residual stresses. The ANN model is capable of determining the correlation between the input variables and corresponding mechanical properties of the weld, from which process parameters can be controlled, monitored, and optimized [12,13,14,15]. Cook et al. uses ANN to model, control, and monitor the variable polarity plasma arc welding, in which a profile analysis of weld bead is performed. Based on the data analysis performed, it is suggested that ANN can give the same real-time results, or even better accuracy and reliability than the previously employed data analysis algorithms [16]. Ismail et al. predicted the weld bead geometry in micro-laser welding using ANN; the algorithm was trained using the backpropagation based on the Levenberg-Marquardt method. The ANN was compared to a mathematical model, and the results indicate that the ANN has better prediction capability than the regression analysis model [17].

The computational efficiency and accuracy of the numerical model can be improved using “the momentum-consistent smoothed particle Galerkin (MC-SPG)” method, proposed by Wu et al. [18]. This is a meshfree approach, in which the unavoidable numerical difficulties encountered during the numerical simulations, which are associated with the meshing issues in modeling the extensive plastic deformation is resolved. Also, a novel velocity smoothing algorithm is introduced to efficiently compute the stress, which does not require stabilization terms. As such, the fundamental difficulty inherent in the stabilization stress computation is avoided [18,19,20,21]. Thus, resulting in much efficient, effective, and accurate computations. The technique has been successfully applied in various numerical simulations of some machining and manufacturing processes, such as grinding [18], thread forming in the flow drill screw-driving process [19], ductile fracture analysis [20] and thermo-mechanical analysis of friction drilling [21].

An accurate prediction of residual stress and deformation resulting from welding is crucial during the manufacturing process of mechanical parts, which improves the reliability of the actual component to be manufactured, limits error, and minimizes manufacturing cost. Therefore, in this paper, a finite element simulation of two pass TIG welding is performed to predict the induced residual stresses and deformation in Al 2219 plate. The FEM is executed using the combination of ABAQUS and FORTRAN user subroutine code. X-ray diffractometer is used to experimentally measure the residual stresses, and the welding induced deformation is examined using a 3-D coordinate measuring machine (CMM) MQ-8106, manufactured by Dongguan Yihui Optoelectronics Technology Co., Ltd, Dongguan, China. Finally, the evolution of residual stress during the two-pass welding was analyzed.

## 2. Materials and Methods

### 2.1. Material

**Figure 1:**
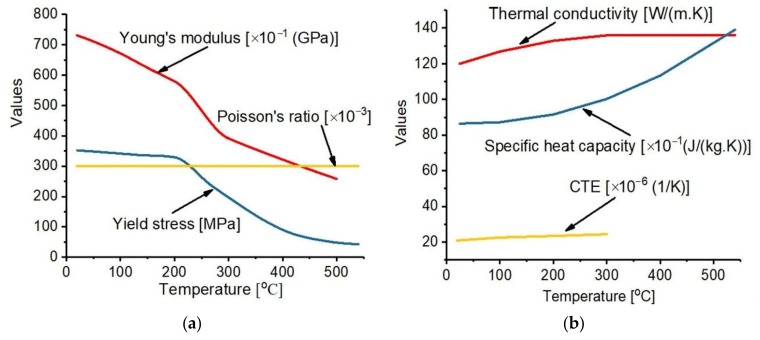
Properties of Al 2219 (**a**) mechanical properties (**b**) thermal properties.

Al 2219 is one of the commonly used materials by the aerospace industries in the manufacture of components such as oxidizers and fuel tanks due to its high strength [22,23] and good fatigue resistance. It is also applied for the manufacture of components operated at a high temperature as well as fabrication of high strength weldments. Al 2219 with an average density of 2840 $\mathrm{kg}/m^{3}$ and composition shown in Table 1 is used in this work, the temperature-dependent properties measured at the Nonferrous Advanced Structure Materials and Manufacturing Research Center, Central South University, Changsha are shown in Figure 1.

### 2.2. FE Modelling and Simulation of TIG Welding

**Figure 2:**
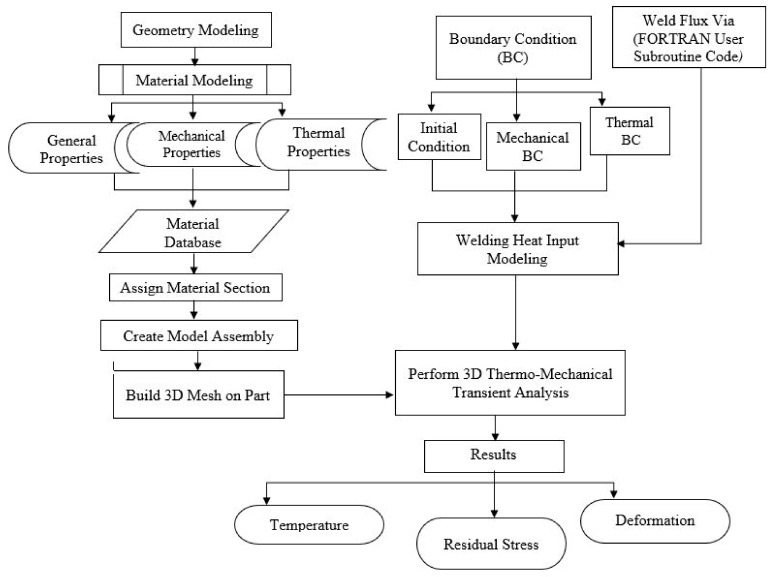
Finite element (FE) simulation process.

Finite element simulation of welding process is a bit complex since it involves thermo-mechanical and metallurgical interactions, as the material is subjected to a differential thermal cycle. The residual stress analysis based on thermo-elastic-plastic (TEP) FEM is usually applied [24,25,26]. Figure 2 illustrates the FE simulation process adopted in this work.

In this paper, a coupled thermo-mechanical, elastic-perfectly plastic FE simulation of TIG welding is performed using the combination of ABAQUS and FORTRAN. The initial and boundary conditions, geometry, and material modeling are established using the ABAQUS, while the heat source model is developed using the DFLUX subroutine code via FORTRAN. In this approach, the magnitude of the nonuniform flux flowing into the model can be defined as body flux or surface flux. The variables included in the DFLUX user subroutine are estimated value of the solution variables (SOL), and the integration point number in the element or surface (NPT), which depends on whether this is a body or surface flux. Also, the step number (KSTEP), the increment number (KINC), the value of step time (TIME), as well as the element number (NOEL) is included in the routine. Additionally, an array containing the coordinates of points (COORDS) is defined, these represent the coordinates of the model if the geometric nonlinearity is considered during the step. Otherwise, the original coordinates of the points are contained in the array [27].

Furthermore, the flux type for the specific DFLUX (JLTYP) is identified, which may be established as a surface-based flux, an element-based flux, or a body flux. Therefore, a body flux is adopted for this model, then all the mentioned variables, together with the Goldak’s heat source governing equations, and the parameters defining the position and displacement of the torch are coded in FORTRAN user subroutine to execute the FE simulation.

**Figure 3:**
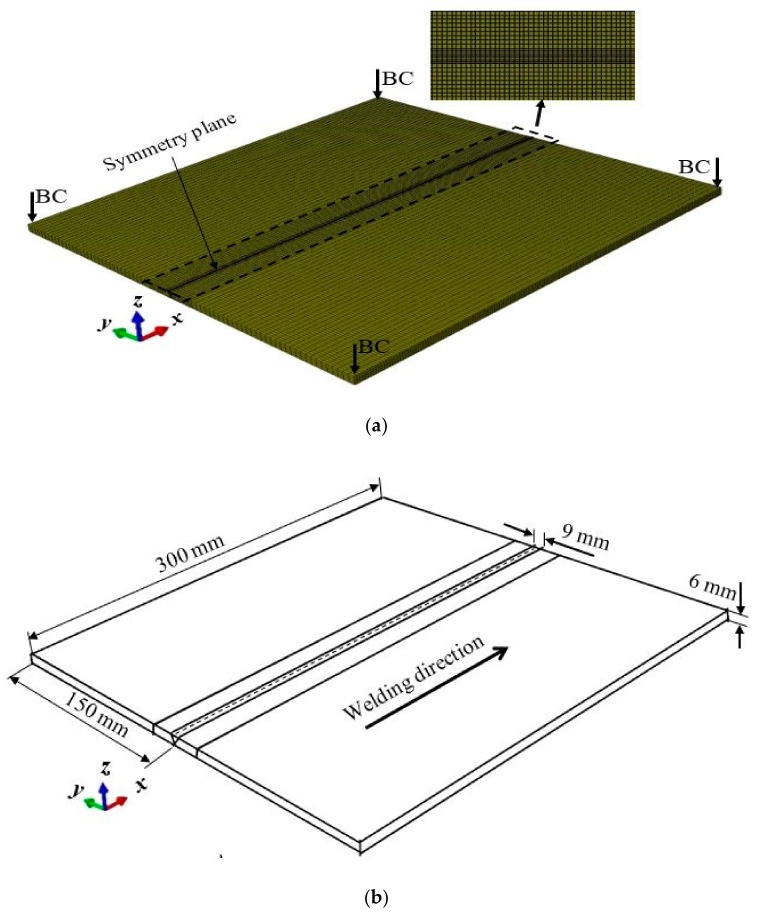
Three-dimensional FE model (**a**) FE mesh (**b**) dimension of the FE model.

Firstly, the thermal analysis is performed, from which the induced thermal field is obtained for every increment, then the mechanical analysis is executed in a sequel as the welding torch passes every node. Thus, resulting in residual stresses and deformation based on the induced thermal load. The three-dimensional FE meshed model of the butt-weld plate is shown in Figure 3, the plate and weld bead are modeled as solid deformable objects in the finite element simulation. The bead which is formed by melting the filler material and weld metal is considered as part of the plate and is the region in which the volumetric heat flux is applied [28]. A fine mesh of element size 0.001 was considered for fusion zone (FZ) and heat-affected zone (HAZ), due to the high-temperature gradient at these regions.

The element type assigned to the FZ and base material is hex C3D8T, the whole FE model contains a total of 81,648 nodes, which is associated with 65,130 elements. Among these, 10,752 nodes and 6680 elements are generated on the fusion zone (FZ) and its vicinity, while the remaining 70,896 nodes and 58,450 elements are assigned to other portions of the plate. The predefined initial temperature of the material is set as 20 °C. Also, the mechanical boundary constraint is applied to the plate to prevent displacement or rotation in all directions but allows deformation during the whole thermal cycle.

#### 2.2.1. Thermal Analysis

**Figure 4:**
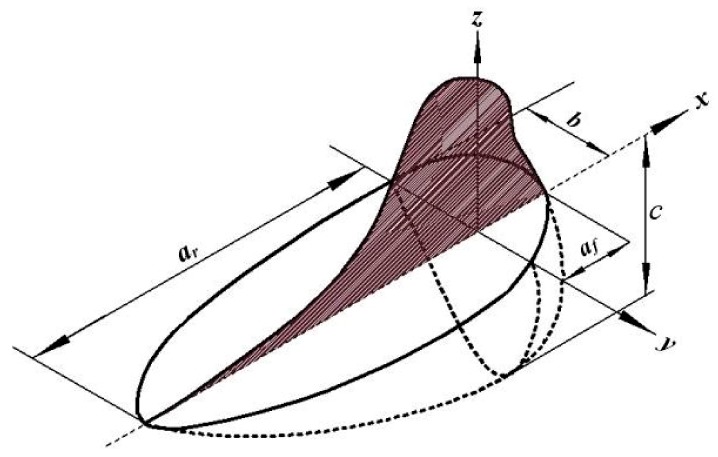
The double ellipsoidal heat source model.

The thermal analysis is based on the transient thermal field induced on the material via the TIG welding arc, which is applied as a volumetric heat source having the front and rear ellipsoidal shapes proposed by Goldak et al. [29]. This heat source model is established with a steep temperature gradient in front of the weld and the slow cooling rate at the rear section of the welding torch due to heat convection in the weld pool [30,31]. Figure 4 illustrates the Goldak’s heat source.

Where $a_{r}$ and $\;\;a_{f}$ represents the rear and front semi-axis of the ellipsoid along *x*, *b* and *c* are semi-axis of the ellipsoid along *y* and *z* respectively. Therefore, the front quadrant’s power density distribution inside the ellipsoid is defined by Equation (1).

(1) $$q_{f}\left(x,y,z,t\right)=\frac{6\sqrt{3}f_{f}Q}{a_{f}bc\pi \sqrt{\pi }}e^{\left(-\frac{3x^{2}}{a_{f}^{2}}-\frac{3y^{2}}{b^{2}}-\frac{3z^{2}}{c^{2}}\right)}$$

Likewise, Equation (2) represents the rear quadrant’s power density distribution inside the ellipsoid.

(2) $$q_{r}\left(x,y,z,t\right)=\frac{6\sqrt{3}f_{r}Q}{a_{r}bc\pi \sqrt{\pi }}e^{\left(-\frac{3x^{2}}{a_{r}^{2}}-\frac{3y^{2}}{b^{2}}-\frac{3z^{2}}{c^{2}}\right)}$$

where *x*, *y*, *z* are the coordinates of the double ellipsoidal heat source, the model parameters $a_{f}\;$, $a_{r}\;$, *b*, and *c* represents the semi-axes of the ellipsoid as described above. *Q* is the net welding power applied to the material, for TIG welding, *Q* is defined as *ηUI*, where *η* is the welding efficiency, *U* (V) is the voltage, and *I* (A) is the current. But *Q* represents the net welding power without considering the additional filler material. Also, the parameters $\;f_{f}$ and $\;f_{r}$ represents the fraction of the heat deposited at the front and rear quadrants during welding [24,28]. The two parameters are described by Equations (3) and (4), where by their sum equals to 2.

(3)  ff=21+afar

(4)  fr=21+araf

**Figure 5:**
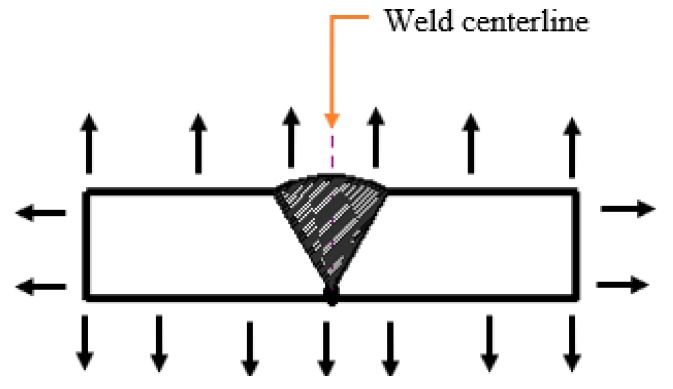
Radiation and convection heat losses in welding.

Heat is transferred to the various sections of the material by conduction as the welding torch moves along the weld centerline (WCL), resulting in non-uniform temperature states. Based on Fourier law, the heat flux *q* (W/m^2^) moves from high to low-temperature regions and linearly dependent on the temperature gradient. Thus, the non-linear transient heat by conduction is governed by Equation (5).

(5) $$\frac{\partial }{\partial x}\left(k\left(T\right)\frac{\partial T}{\partial x}\right)+\frac{\partial }{\partial y}\left(k\left(T\right)\frac{\partial T}{\partial y}\right)+\frac{\partial }{\partial z}\left(k\left(T\right)\frac{\partial T}{\partial z}\right)+Q=\rho \left(T\right)C_{p}\left(T\right)\frac{\partial T}{\partial t}$$

where $C_{P}$ denotes the specific heat capacity of Al 2219, *ρ* is the density, *k* is the isotropic thermal conductivity, *T* is the temperature, and *Q* is the welding heat input. Solving the problem in solidus and liquidus domain by treating the heat diffusion with an enthalpy-based formulation, the heat transfer governing equation becomes:

(6) $$\begin{array}{ll} \rho \left(T\right)C_{p}\left(T\right)\frac{\partial H}{\partial t}= & \left[\frac{\partial }{\partial x}\left(\frac{k\left(T\right)}{\rho \left(T\right)C_{p}\left(T\right)}\;\frac{\partial H}{\partial x}\right)+\frac{\partial }{\partial y}\left(\frac{k\left(T\right)}{\rho \left(T\right)C_{p}\left(T\right)}\;\frac{\partial H}{\partial y}\right)+\frac{\partial }{\partial z}\left(\frac{k\left(T\right)}{\rho \left(T\right)C_{p}\left(T\right)}\;\frac{\partial H}{\partial z}\right)\right]\\ & +Q \end{array}$$

where *H* is the enthalpy. The thermal boundary conditions taken into account during the thermal analysis are the heat losses by radiation and convection to the environment. Even though there is a fraction of heat loss by conduction due to mechanical restraint, but it’s mostly neglected in the majority of research works. Equation (7) represents the heat loss by radiation.

(7) $$\frac{dQ}{dt}=\varepsilon_{r}\cdot \sigma_{B}\cdot A\left(T^{4}-T_{c}^{4}\right)$$

where $\;\varepsilon_{r}$ is the Al 2219 emissivity, *A* is the surface area considered for radiation, $\sigma_{B}$ is the Stefan Boltzmann constant, *T* is the material’s temperature and, $T_{C}$ is the temperature of the surrounding. Also, the heat loss by convection to the environment is defined by Equation (8).

(8) $$\frac{dQ}{dt}=h\cdot A\left(T-T_{c}\right)$$

where *h* is the coefficient of heat transfer by convection. Taking the effect of clamping into consideration, the heat transfer between the workpiece and the attached clamping devices can be expressed by Equation (9).

(9) $$\frac{dQ_{h}}{dt}=k\cdot A\left(T-T_{clamp}\right)$$

where *k* is the heat transfer coefficient between the clamps and the workpiece, and $T_{clamp}$ is the temperature of the clamps. Figure 5 illustrates the thermal radiation and convection taking place during welding.

The thermal boundary conditions stated in the thermal analysis are fully taken into consideration in the FE simulation, except the heat loss due to mechanical restraint. The convection coefficient, material emissivity, and Boltzmann constant are $h=15\;{\mathrm{Wm}}^{-2}\;K^{-1}$, *ε* = 0.7 [32], $\sigma_{B}=5.669\times 10^{-8}{\;\mathrm{Wm}}^{-2}K^{-4}$ [33] respectively. The welding conditions and parameters applied in the FE simulation are shown in Table 2.

#### 2.2.2. Mechanical Analysis

The mechanical analysis is based on the fundamental principles of thermal-elastic-plastic equations. During welding, the material is strained as a result of the weld thermal cycle, the total strain $\varepsilon_{total}$ which includes the components of elastic strain $\varepsilon_{e}\;$, thermal strain $\varepsilon_{T}\;$, plastic strain $\varepsilon_{p}\;$, volumetric strain $\varepsilon_{V}\;$, and transformation-induced plastic strain $\varepsilon_{Tr}$ results in the residual stresses and deformation [34]. The stress and strain can be related using the generalized Hook’s law for an isotropic material [35]. The induced plastic deformation is associated with Von mises criterion, whereby the equivalent stress is defined by Equation (10) [7].

(10) $$\sigma_{v}=\sqrt{\frac{1}{2}\left[{\left(\sigma_{1}-\sigma_{2}\right)}^{2}+{\left(\sigma_{2}-\sigma_{3}\right)}^{2}+{\left(\sigma_{3}-\sigma_{1}\right)}^{2}\right]}$$

where $\sigma_{1}\;$, $\sigma_{2}\;$, and $\sigma_{3}$ are the principal stresses, and the total strain is defined as the sum of individual strain components such that:

(11) $$\varepsilon_{total}=\varepsilon_{e}+\varepsilon_{p}+\varepsilon_{T}+\varepsilon_{V}+\varepsilon_{Tr}$$

Neglecting the effects of volumetric change strain and transformation-induced plastic strain, the stress field $\left(\sigma_{e}\right)$ and displacement field ($U_{e}$) can be determined using Equation (12) by combining the equilibrium and constitutive equations.

(12) $$\left\{K_{1}\right\}\left\{U_{e}\right\}-\left\{K_{2}\right\}\left\{T_{e}\right\}=\left\{R\right\}$$

where $\left\{T_{e}\right\}$ is the temperature field, $\left\{U_{e}\right\}$ displacement field, $\left\{K_{1}\right\}$ and $\left\{K_{2}\right\}$ are the stiffness matrix and {R} is the temperature loads of each node.

### 2.3. Experimental Validation

**Figure 6:**
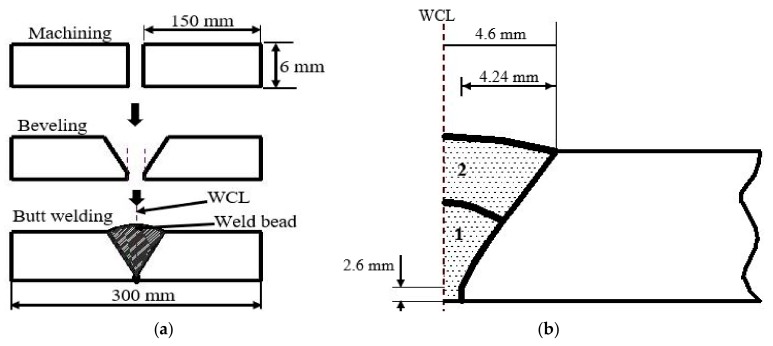
Experimental sequence. (**a**) the machining and manufacturing processes; (**b**) description of the weld-passes and groove dimension.

The TIG welding experiment is carried out to validate the FE model, two pieces of Al 2219 plates with dimension 300 mm × 150 mm × 6 mm each were butt-welded using TIG welding. The parallel edges to be joined were beveled prior to the welding; the two chamfered edges formed a groove when aligned which accommodate the filler materials. Residual stress as high as 186.3 MPa were observed at the machined regions after the beveling operation. This can lead to hot cracking and high deformation of the plate during welding and can also result in crack propagation at ambient temperature, which increases its vulnerability to failure. Therefore, the specimens were heat-treated by heating to an elevated temperature of 500 °C and held for 2 h, then cooled uniformly in the furnace to ambient temperature. The post-machining heat treatment significantly relieved the induced residual stresses to an accepted level. Figure 6 illustrates the experimental sequences carried out.

**Figure 7:**
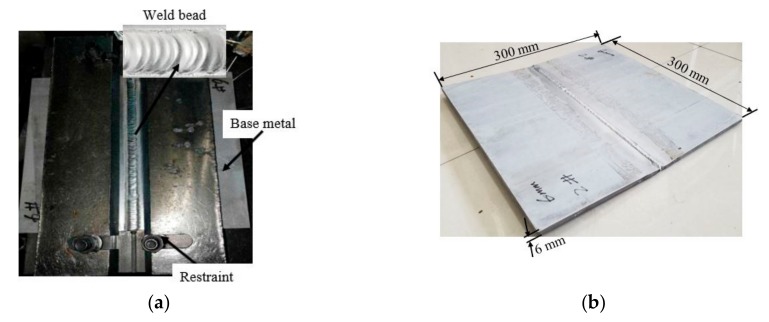
As-weld workpiece (**a**) specimen with restraint (**b**) as-weld Al 2219 plate.

The workpiece is then butt-welded in a double pass with filler material ER70S-6 (Ø 1.6 mm) which fused in the machined groove on the workpieces forming the weld bead as shown in Figure 7. The weld zone is protected from oxidation and impurities using an argon shielding gas. The welding duration for each pass was recorded from which the speed (*v*) was computed. The current *I* (A), voltage *U* (V), and efficiency (*η*) were also recorded, and the heat input *q* is calculated. Thus, the heat input per every 1mm length of weld ($Q_{W}$) is determined using Equation (13) [28]. The welding parameters are shown in Table 3.

(13) $$Q_{w}=\frac{IU\eta }{v}$$

### 2.4. Residual Stress Measurement Using X-ray Diffractometer (XRD)

**Figure 8:**
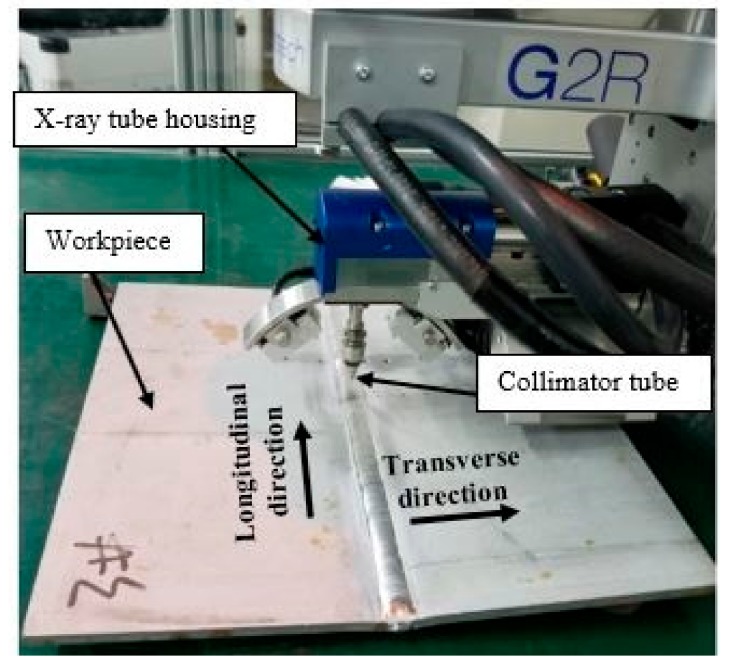
Residual stress measurement using XRD.

The residual stresses induced due to the weld thermal cycle were experimentally analyzed and are compared to the FEM’s to validates the FE model. X-ray diffractometer (Xstress 3000 G2/G2R, X-ray Stress Analyzer, manufactured by Stresstech Bharat Pvt Ltd., Mumbai, India) was used to measure the stresses. Table 4 presents the diffractometer parameters used. Figure 8 shows the residual stress measurement using XRD. Only the near-surface stresses can be examined using this technique. Therefore, the comparison was made for only the surface stresses. The measurement was carried out in steps of 5mm interval in the transverse direction, and 30 mm interval along the longitudinal direction of the weld bead.

### 2.5. Measurement of Welding Deformation Using Coordinate Measuring Machine (CMM)

**Figure 9:**
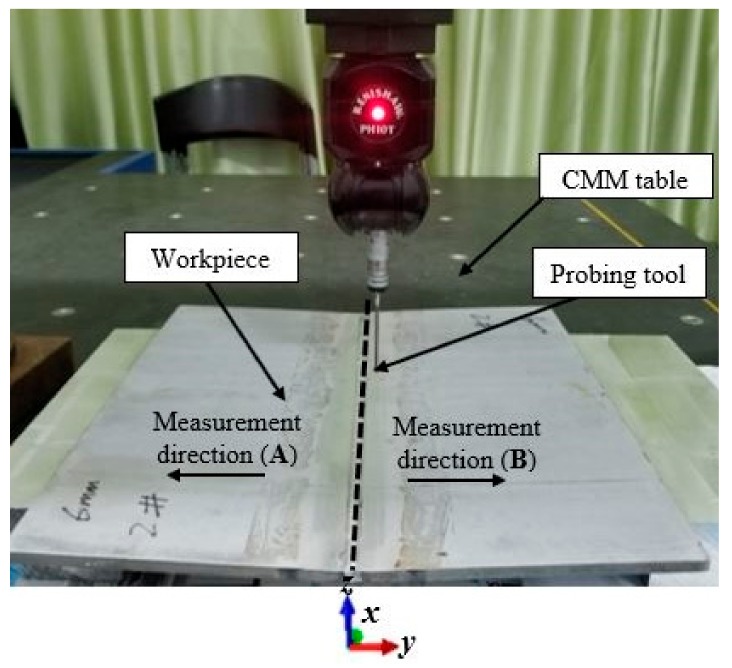
Experimental measurement welding distortion.

The induced deformation on the weld plate is measured using a three-dimensional coordinate measuring machine (3-D CMM) MQ-8106. The measurement was carried out before and after the welding. Fourteen selected points from each section of the plate (A and B) were measured along the transverse direction of the weld plate in steps of 10 mm interval, and the displacements along z-axis were recorded. The experimental measurement of the induced deformation is shown in Figure 9.

## 3. Results and Discussions

### 3.1. Thermal Field Induced by the Two-Pass Butt-Welding

**Figure 10:**
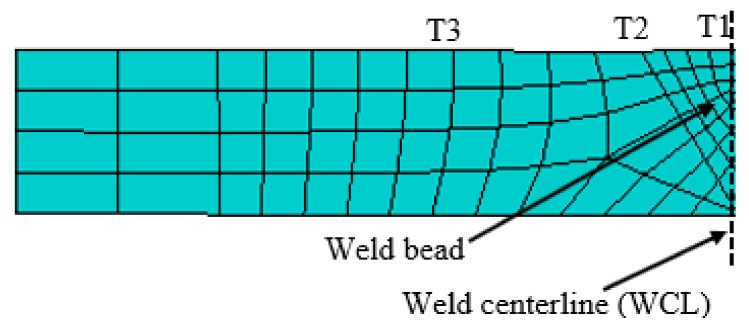
Thermal history positions.

The temperature history from the FE simulation was extracted at three different positions (T1, T2, and T3) on the weld model, at 150 s and 450 s for pass 1 and 2 respectively. The first position being the heat source location along the WCL, then, 5 mm and 10 mm away from the WCL as shown in Figure 10.

**Figure 11:**
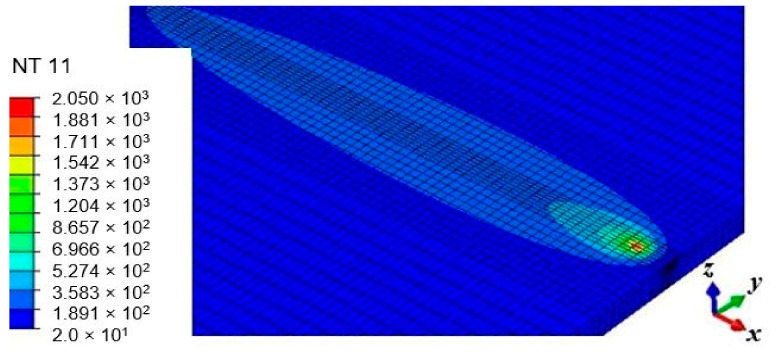
Thermal distribution.

**Figure 12:**
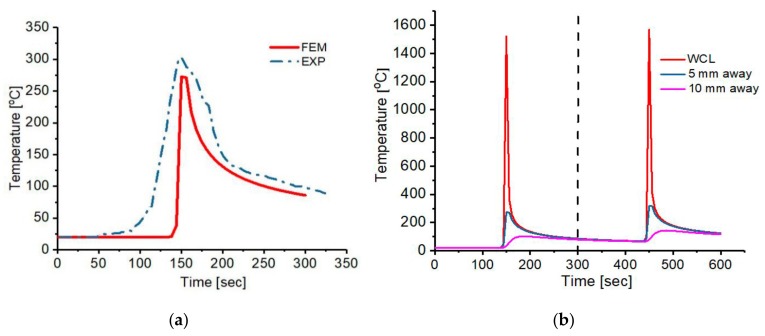
(**a**) Compared experimental and FEM temperature history at 5 mm away from the heat source (**b**)The predicted temperature history of various positions during the first and second pass.

The temperature distribution on the plate from the FE simulation is shown in Figure 11. Also, the temperature histories are shown in Figure 12. Position T1 attained the peak temperature of 1521.54 °C and 1567.75 °C after pass 1 and 2 respectively. An inter-pass temperature of 86.76 °C was determined between pass 1 and 2, but an inter-pass cooling effect was not simulated in this work. The consequences of non-uniform cooling due to convection and radiation results in a rapid drop of the peak temperature as the heat source passes a particular position with constant speed, which in turns leads to residual stress formation. Also, point T2 reaches the peak temperature of 272.54 °C and 318.92 °C during pass 1 and 2 respectively. In addition, T3 attained the highest temperature of 101.60 °C during pass 1 as well as 143.28 °C in pass 2. The heat deposited is transferred to the low-temperature regions of the plate by conduction and loss to the surrounding environment by radiation and convection, as mentioned earlier.

### 3.2. Comparison of Residual Stresses Between FEA and Experiment

**Figure 13:**
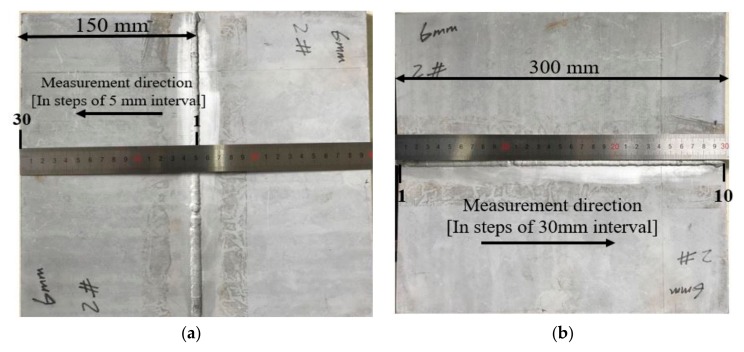
Residual stress measurement location and direction (**a**) residual stress measurement in transverse direction (**b**) residual stress measurement in longitudinal.

Peak tensile residual stress of 371.7 MPa was observed at weld zone (0 mm away from the bead), which decreases continuously as the distance away from weld zone increases, it then attains the highest compressive stress of −138.4 MPa around the vicinity of HAZ (15 mm away from bead). Furthermore, a significant decrease in the magnitude of the stress was observed at a position around 50 mm away from the bead, in which −8.6 MPa and 2.2 MPa were determined at 50 mm and 55 mm away from the weld bead. Moreover, a further increase in the residual stresses was observed whereby 28.3 MPa to 52.8 MPa tensile stresses were observed between 60 mm and 120 mm away from the weld bead. This can be as a result of the restraint positioning because the middle and edges of the joined plates were not well constrained, which affects the distribution of residual stress. Also, the magnitude and behavior of the specimen’s deformation are affected to a certain degree. The positions in which the residual stress measurement was carried out are shown in Figure 13.

**Figure 14:**
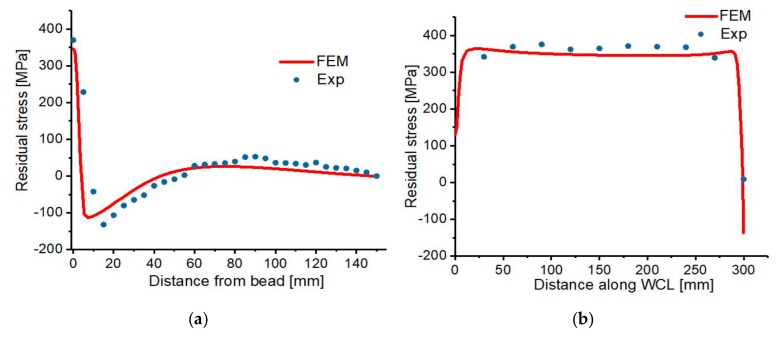
(**a**) Residual stresses in the transverse direction (**b**) residual stresses in the longitudinal direction.

Also, based on the FEM results, the highest tensile and compressive residual stresses at the surface of the model are 346.321 MPa and −113.623 MPa respectively. But this is not the peak residual stress induced in the whole model, the stress in the core region is as high as 489.4 MPa, which cannot be compared with the experimental because the core stresses cannot be analyzed experimentally. Figure 14 shows the compared surface transverse and longitudinal residual stress; the results show good agreement with 18.8% variation.

### 3.3. Residual Stress Evolution

**Figure 15:**
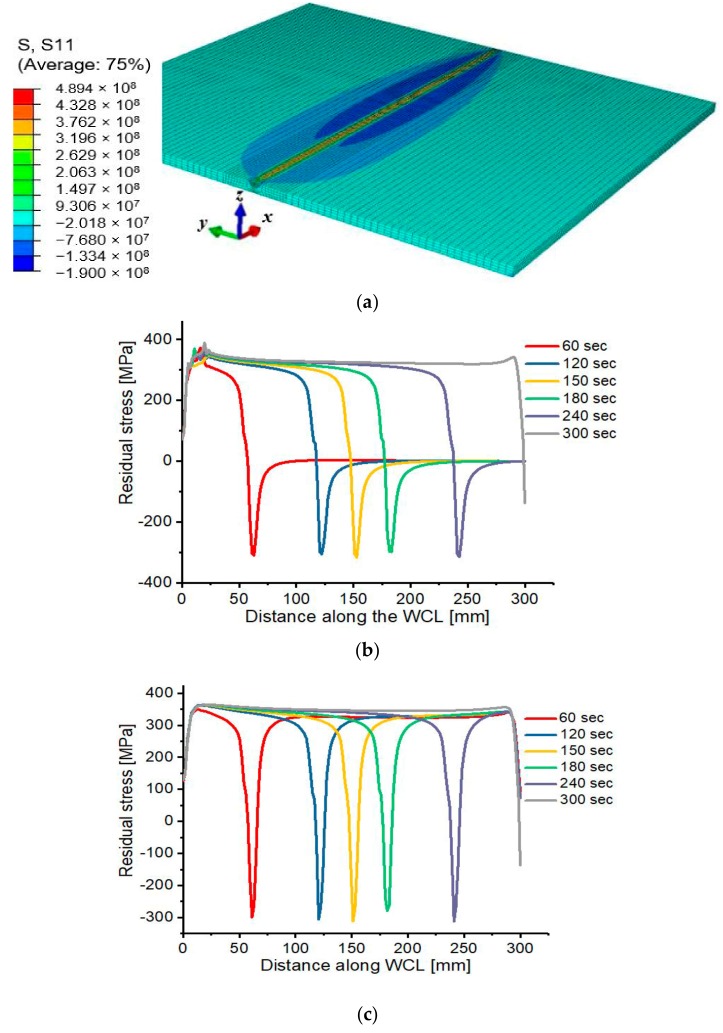
(**a**) Residual stress distribution (**b**) Residual stress evolution during the first pass (**c**) Residual stress evolution during the second pass.

**Figure 16:**
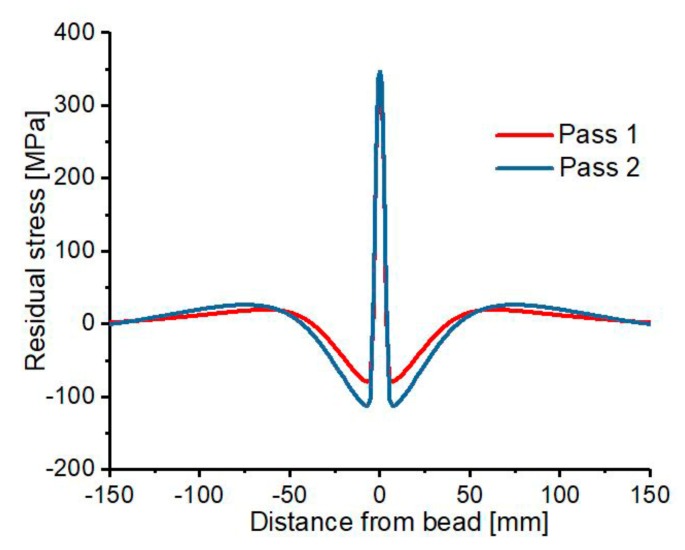
Residual stress distribution in the transverse direction.

Different temperature states during the first and second weld-pass results in volumetric and strength changes in the various positions on the weld plate, which depends on the heat source location. The movement of the heat source along WCL gives rise to the variation in temperature, residual stresses, and deformation at different regions of the Al 2219 plate. The temperature of any position along the WCL increases as the heat source move close to it, resulting in variation of residual stress’s magnitude and distribution. As shown in Figure 15, the evolution of residual stress is examined after every 60 s to evidently analyze its distribution during the whole welding process. Initially, the material is free of stresses, but as the heat source moves for 60 s during the first pass, compressive residual stress of −309.143 MPa develop at the corresponding heat source position due to increase in thermal expansion at the vicinity of weld metal being heated for melting, and at the same time, peripheral low-temperature region restrained the thermal expansion. After reaching peak value, a gradual decrease in the compressive residual stress take place due to the softening of the weld zone and finally reduces to zero. Then, as the heat source move for 120 s, the previously welded positions begin to solidify, resulting in high tensile residual stress of up to 371.542 MPa at the welded areas. The process is repeated continuously throughout the welding process, in which the final residual stress distribution is obtained after 300 s, and a peak tensile stress of 388.39 MPa, and peak compressive stress of −186.8 MPa are induced along the weld zone. The same scenario is observed during the second pass, in which a maximum tensile and compressive residual stresses of 489.4 MPa and −190 MPa were determined respectively along the weld zone after the second pass is completed. The nature of evolution and distribution of residual stresses is the same for the first and second pass but differ in magnitude as presented in Figure 16.

### 3.4. Comparison Between FEA and Experimental Distortion

**Figure 17:**
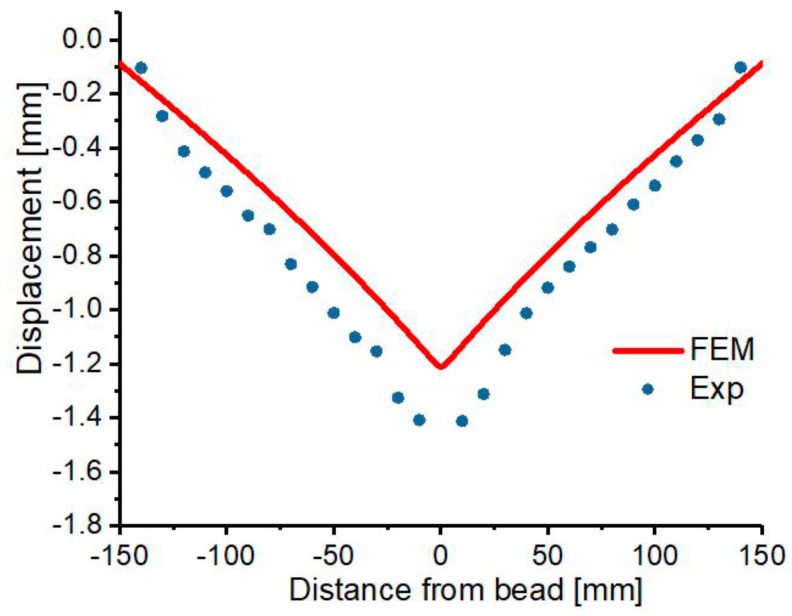
Compared welding distortion.

The highest experimental and FEM deformation along the z-axis is −1.791 mm and −1.2114 mm respectively, which is determined around the weld zone. The compared results are shown in Figure 17, the highest and lowest variation in the result is 32.4% and 9.94% respectively. The large deviation of 32.4% is determined at the region with peak distortion. This is as a result of variation in mechanical restraint positioning, which is a very important parameter that offers resistance to material’s deformation during cooling of welds. The observed distortion is significant that it may require repair, rework and will lead to a reduction in the aesthetical value of the workpiece, thus, increasing manufacturing cost.

**Figure 18:**
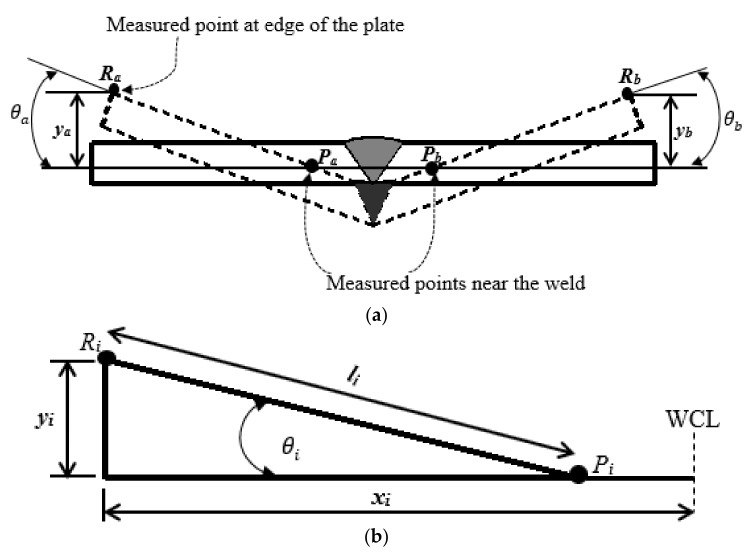
Schematic diagrams for computing the angular distortion. (**a**) distorted weld metal; (**b**) extracted measured locations for determining the corresponding angular distortion.

Figure 18. Illustrates the examined positions near the weld zone and edges, which can be related to evaluate the resulting angular distortion from both side of the plate (side a and b).

As illustrated in Figure 18a,b, $y_{i}$ is the measured z-displacement at the edge, $l_{i}$ is the length from the point measured near the weld $\left(P_{i}\right)$ and point measured at the edge $(R_{i}\;$), $\;x_{i}$ is half width of the plate and $\theta_{i}$ is the resulting angular distortion, *a* and *b* identifies the side in which the measurement was carried out. Therefore, the angular distortion can be determined using Equation (14).

(14) $$\theta_{i}=\sin^{-1}\left(\frac{y_{i}}{l_{i}}\right),\;\;\;\;i=a,b$$

The distance $l_{i}$ is 140 mm, then, the calculated angular distortions from each side of the plate ($\theta_{a}$) and ($\theta_{b})$ are $0.79^{{}^{\circ}}$ and $0.79^{{}^{\circ}}$ respectively.

### 3.5. Welding Deformation and Shrinkage

**Figure 19:**
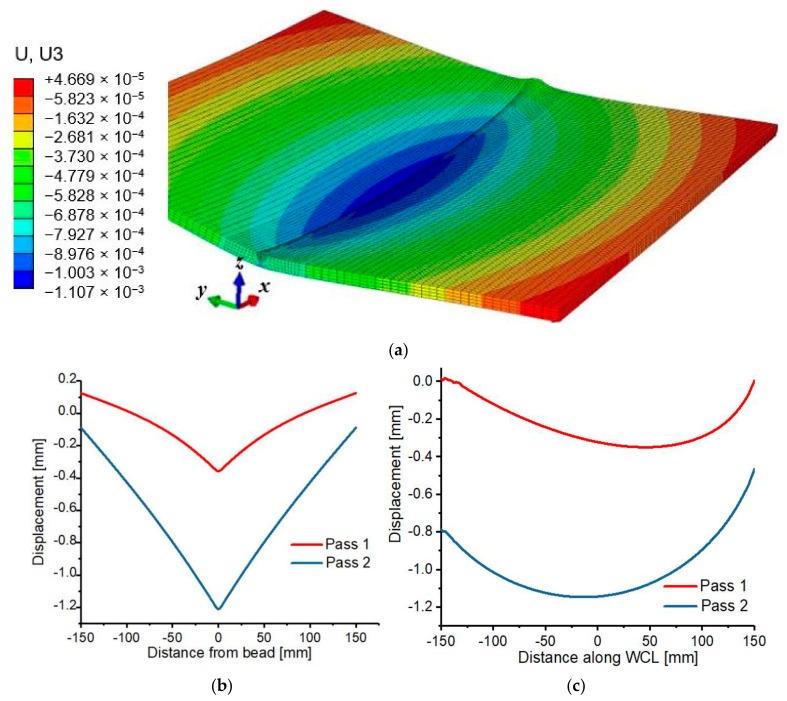
(**a**) FE welding distortion (**b**) Transverse distortion (**c**) Longitudinal distortion.

The material plastically deforms and shrink due to gradual non-uniform drop in temperature as the heat source passes a specific position along the WCL. The continuous temperature drops during cooling results in continuous shrinkage of the hot base material and HAZ, in which at a certain point, the material become strengthen, thus, developing resistance to shrinkage due to low temperature. As such, the weld plate remains in strained condition due to this behavior of contraction. Generally, material shrinkage is significantly affected by the variation in thermal expansion between the weld zone and low-temperature base metal at the cooling phase of the thermal cycle. The FE welding deformation induced by pass 1 and 2 along the longitudinal and transverse direction is shown in Figure 19. The peak deformation is −1.2114 mm, which is observed at the weld zone, then gradually decrease in the transverse direction and attained a minimum value of 0.04669 mm at the edge of the plate. The peak deformation observed at the weld zone results from the high plastic deformation experienced by the region during the thermal cycle.

**Figure 20:**
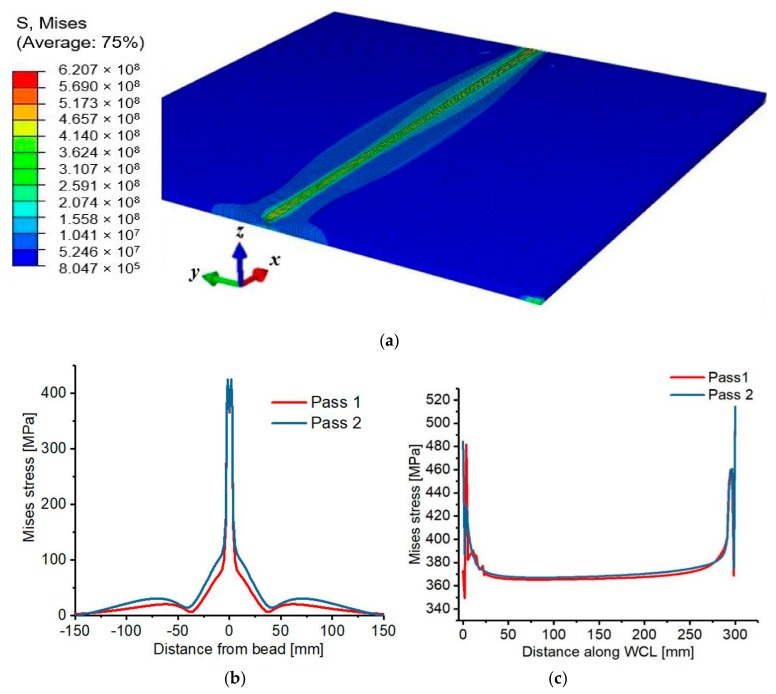
(**a**) Von Mises residual stress distributions (**b**) transverse stresses (**c**) longitudinal stresses.

Furthermore, parameters such as the number of weld-passes, welding speed, time interval between weld-passes as well as the intensity and position of mechanical boundary constraint significantly influence the magnitude and distribution of residual stresses and distortion. Figure 20 shows the Von Mises stress based on pass 1 and 2.

Generally, the difference in residual stresses and deformation induced by pass 1 and pass 2 arise from the variation of yield stress, elastic modulus, thermal conductivity, coefficient of thermal expansion and heat transfer coefficients of the material after the thermal cycle of the first and second pass. High coefficient of thermal expansion and yield strength leads to high residual stress.

If not carefully performed, multi-pass welding can lead to hot cracking of welds and high deformation due to the multiple thermal cycles induced on the material. The peak inter-pass temperature must also be controlled, which should be less or equal to the maximum allowable for the material under consideration. Therefore, all the welding parameters stated earlier must be given attentive consideration during multi-pass welding.

## 4. Conclusions

A 3-D FE model developed using ABAQUS 2017 and FORTRAN user subroutine code to predict the residual stresses and deformation induced by two-pass welding of Al 2219 plate is presented in this paper. The DFLUX subroutine code was established to represent the distribution power density of the moving weld torch based on Goldak’s double ellipsoidal heat source model. The effects of convection and radiation heat losses have been taken into account. Also, the welding-induced residual stresses and deformation based on the FE model have been validated experimentally. Furthermore, the effects of the number of weld-passes on residual stress and distortion were analyzed. Based on the obtained results in this work, it can be concluded that:

(1) Based on the experimental validation performed, the implemented FE model is feasible and reliable for computing the thermal field, residual stresses, and deformation induced by two-pass TIG welding on Al 2219 plate.
(2) A very high residual stress which is up to 0.788 of the Von Mises stress and 0.81 of the material’s yield strength is induced at the vicinity of the weld zone, consequently resulting in the noticeable deformation observed. The residual stress is so high that reduction is necessary to enhance the mechanical performance of the weld plate.
(3) The observed experimental distortion is significant that the plate may require repair or rework, thus resulting in higher manufacturing cost.
(4) High inter-pass temperature can develop on the workpiece from the previous pass, which may lead to hot cracking of weld during the subsequent passes. Therefore, an inter-pass cooling is recommended after completing each weld pass.

## Figures and Tables

**Table 1:** Al 2219 composition.

Weight %	Al	Si	Fe	Cu	Mn	Mg	Zn	Ti	Others
Al 2219	Balance	0.20Max	0.30Max	5.80–6.80	0.20–0.40	0.02 Max	0.10 Max	0.02–0.10	Max 0.050 each Max 0.15 total

**Table 2:** Heat source parameters for the FE simulation.

Parameters	Values
Efficiency (*η*)	0.7
Current (*I*)	12 A
Voltage (*U*)	200 V
*a_f_*	6.0 mm
*a_r_*	12 mm
*b*	3.5 mm
*c*	5.0 mm
*f_f_*	1.33
*f_r_*	0.67

**Table 3:** TIG welding parameters.

Parameters.	Values
Efficiency (*η*)	0.7
Current (*I*)	12 A
Voltage (*U*)	200 V
Ambient Temperature	19 °C
Welding Speed (pass 1)	2.6 mm/s
Welding speed (pass 2)	2.8 mm/s
$Q_{W}\;$(pass 1)	0.646 ${\mathrm{kJmm}}^{-1}$
$Q_{W}\;$(pass 2)	0.600 ${\mathrm{kJmm}}^{-1}$

**Table 4:** XRD measurement parameters.

X-ray Diffraction Parameters	Specification/Values
Tube type	Cr
Max. Current	9 A
Max. Power	270 W
Supplied current during the experiment	6.7 A
Supplied voltage during the experiment	30 V
Exposure time for the calibration	8 s
Exposure time for measurement	10 s
Collimator diameter	3 mm
Collimator distance	10.390 mm
Detector distance	50 mm
Tilt angle	−45° to 45°
Number of tilts	5/5
Rotation angle	0° to 90°
Number of rotations	2
